# Mechanical Ventilation for Comatose Patients with Inoperative Acute Intracerebral Hemorrhage: Possible Futility of Treatment

**DOI:** 10.1371/journal.pone.0103531

**Published:** 2014-07-25

**Authors:** Toru Fukuhara, Mizuho Aoi, Yoichiro Namba

**Affiliations:** Department of Neurological Surgery, National Hospital Organization Okayama Medical Center, Okayama, Japan; The Hospital for Sick Children and The University of Toronto, Canada

## Abstract

**Background:**

Comatose patients with acute intracerebral hemorrhage (ICH) diagnosed as inoperative due to their severe comorbidity will be treated differently between countries. In certain countries including Japan, aggressive medical care may be performed according to the patients' family requests although the effects on the outcome are obscure. For respiratory distress in comatose patients with inoperative acute ICH, the role of mechanical ventilation on the outcome is unknown. We speculated that the efficacy of a ventilator in such a specific condition is limited and possibly futile.

**Methods:**

We retrospectively evaluated the in-hospital mortality and further outcome of 65 comatose patients with inoperative ICH. Among the patients, 56 manifested respiratory distress, and the effect of the ventilator was evaluated by comparing the patients treated with and without the ventilator.

**Results:**

The in-hospital mortality was calculated as 80%. A statistically significant parameter affecting the mortality independently was the motor subset on the Glasgow Coma Scale (*P* = 0.015). Among the patients who manifested respiratory distress, 7.7% of patients treated with a ventilator and 14.0% of patients not treated with a ventilator survived; an outcome is not significantly different. The mean survival duration of patients treated with a ventilator was significantly longer than the mean survival duration of patients not treated with a ventilator (*P* = 0.021). Among the surviving 13 patients, 7 patients died 5 to 29 months after onset without significant consciousness recovery. Another 6 patients suffered continuous disablement due to prolonged severe consciousness disturbances.

**Conclusion:**

The current results indicate that treating comatose patients resulting from inoperative acute ICH may be futile. In particular, treating these patients with a ventilator only has the effect of prolonging unresponsive life, and the treatment may be criticized from the perspective of the appropriate use of public medical resources.

## Introduction

The medical care limitations associated with the do not resuscitate (DNR) order at the acute stage of intracerebral hemorrhage (ICH) is a well-recognized independent factor leading to poor prognosis [1]–[3]. In spite of the definition of a DNR order: “no attempt of resuscitation required in the case of cardiopulmonary arrest”, a DNR obtained early leads to an overall lack of aggressive care [2]. A DNR order does not necessarily mean the withdrawal of medical efforts leading to the best possible outcome. In fact, it was reported that proper medical care even with DNR orders did not lead to a poor outcome of patients with ICH [4].

However, medical resources are limited in the rapidly aging society, and since medical resources are public in nature, the priority for using limited medical resources should be seriously discussed. This “medico-social triage,” involving treatment priorities even in a non-disaster situation, will be the physicians' responsibility in the near future, in order to maximize the quality and quantity of cured lives. Especially in Japan where even the brain-dead patients are commonly managed with continuous medical care according to their family's wishes, the recognition of medical futility is the first important step to keep the medical care system functioning properly.

For the purpose of decreasing the burden on medical resources, medical care termination as early as possible has a larger impact, although early outcome prediction is frequently difficult [5], [6]. In the management of ICH, the role of surgical hematoma evacuation is multifactorial and it has been difficult to establish simple guidelines [7], [8]. Comparatively, the patient's condition with inoperative ICH would be less complicated and the treatment strategy limited. Although artificial support with a mechanical ventilator might be the choice in the case of respiratory distress, it often sustains unresponsive life leading to eventual death. Many patients' families refuse ventilator support to avoid sustaining unresponsive life. However, there exists a dilemma in making a decision regarding the uncertainty whether comatose patients due to ICH with respiratory distress have a chance to recover, and if they do recover, how significant their functions would be.

The aim of this study is to evaluate the efficacy of mechanical ventilator support on the outcome of comatose patients with inoperative ICH. As well as determining the factors affecting the in-hospital mortality for these patients, the effects of a ventilator on sustaining life and the final outcome are further explored. Based on these results, medical futility in neurosurgical fields is extensively discussed.

## Materials and Methods

### Study Population

After obtaining approval by the Institutional Review Board of National Hospital Organization Okayama Medical Center in December 2012 (H24-59), the medical records of the patients who were admitted to our hospital due to non-traumatic spontaneous ICH between July 2006 and June 2013 were retrospectively reviewed. According to the ethical guideline for clinical research, published by the Japanese Ministry of Health, Labor and Welfare (revised version on July 31, 2008), the retrospective clinical study without involving the analysis on biological samples from the patients does not require obtaining the informed consent, but does require notifying the information about the study, including the purpose and method, described on page 19 (in Japanese). Since this study is the retrospective study without utilizing any biological samples, obtaining the informed consent is not required, and no informed consent was taken for this retrospective observational study. Instead, the information of the study has been open to public through the homepage of the hospital and posting a notice in the hospital. Any identifiable information of the patients including name, birthday, address and the admission or discharge date were not extracted for the study, in order to anonymized the data prior to analysis.

During these 7 years, 471 patients (8 patients were counted twice because of different admissions with two distant attacks) were admitted to our hospital due to ICH without known organic etiologies. Among these patients, comatose patients, defined as both subsets of the Glasgow Coma Scale (GCS) on the verbal response and on the eye opening response scored at 1, diagnosed at admission or deteriorated to coma within 24 hours after admission, were extracted. In order to exclude epileptic deterioration with acute ICH onset, patients who improved within 48 hours after admission were not counted. After the exclusion of the patients to whom emergency hematoma evacuation surgery was offered, there remained 66 patients whose hematomas were considered to be inoperative due to their associated conditions. Inoperative conditions were classified into 6 categories: 1) high age over 90 years old (8 patients), 2) unstable vital signs and highly likely to die during surgery (11 patients), 3) disturbances in hematological laboratory data; defined as a platelet count of less than 80,000 and/or INR>2.5 (20 patients), 4) associated with terminal uncontrolled diseases (7 patients; 2 patients with liver cirrhosis, 2 patients with multiple myeloma, 1 patient with myelodysplasia, 1 patient with gastric cancer and 1 patient with lung cancer), 5) associated with severe active diseases (6 patients; 2 patients with acute myocardial infarction, 1 patient with severe liver dysfunction, 1 patient with progressing untreated renal failure, 1 patient with severe uncontrolled diabetes and 1 patient with lung emphysema) and 6) hematoma location on the brain stem, either primary or extending into (38 patients). Emergency surgery was not offered for these 66 patients, since they had at least one of the inoperative conditions mentioned above. One patient whose family wished discontinuation of the dialysis and died 2 days after admission was excluded from the analysis, since the mortality of this patient was strongly affected by the withdrawal of the dialysis. Patients whose dialysis continued after the admission or who died within 2 days after the admission were included so the analysis was performed with 65 patients. When the patients were comatose at admission or deteriorated to coma after the admission, we asked their families whether treatment with a mechanical ventilator was preferred in case of respiratory distress occurrence. We explained the effects of ventilators as, “it mostly sustains life for several days, but it may increase the survival rate. Surviving patients are most likely to be in a vegetative state.” Families of 48 patients refused treatment with a mechanical ventilator for the patients, and the patients were managed without a ventilator in spite of 43 patients progressing to respiratory distress. Families of 9 patients required the patients to be managed with a ventilator, and 7 patients were actually managed with a ventilator, although 2 patients did not progress to respiratory distress. In 2 patients, the families were unable to decide, however respiratory distress did not occur to these patients, and they were managed without a ventilator. In 6 patients, treatment with a ventilator was initiated in the emergency room (ER) according to the regular resuscitation protocol before confirming the families will, so 13 patients in total, due to the families' requirement in 7 patients and due to the resuscitation in the ER in 6 patients, were managed with a ventilator.

### Management Protocol

With diltiazem hydrochloride infusion, maintaining a systolic blood pressure of less than 150 mmHg was the first target of the treatment. In order to decrease the intracranial pressure, 20 g glycerol in 5% fructose was infused three times per day. For supratentorial hematoma, phenytoin infusions were initiated to avoid epileptic deterioration. Associated significant disorders were also treated; suspected infections such as pneumonias or urinary tract infections were treated with antibiotic infusions. Vitamin K injection was added in the case of INR>1.5. No patients became “operative” after the reversal of the coagulopathies, since other associated conditions prevented the patients from requiring evacuation surgeries (mostly too late with unstable vital signs).

Continuous monitoring of arterial oxygen saturation with transcutaneous pulse oximetry digit sensor was performed immediately after arriving at the hospital and high-flow oxygen supply up to 6 liters per minute with non-rebreather mask was initiated at SpO2 of less than 98%. Upper airway obstruction due to coma by the tongue root was treated initially with nasal cannula, and in selected cases with severe obstruction, endotracheal intubations were performed. We seldom used sedative medication for comatose patients with ICH because the usage disturbed the exact evaluation of the consciousness level. Respiratory distress, such as frequent apnea of more than 10 seconds, or ataxic breathing was the indication of the ventilator, and ventilator support was provided to patients whose families preferred that option. Any doubtful respiratory abnormalities were confirmed with an arterial blood gas examination. Other indications of the ventilator for patients with ICH include; intravenous sedative medication usage for controlling the pain after surgery or status epilepticus, and pulmonary diseases requiring ventilation support irrespective of the consciousness level, both of which were not included in the studied patient cohort. During the ventilator support, the treatment was continued, and the blood pressure reduction at the terminal stage was treated with continuous dopamine infusion. In our institution, as is common in Japan, the diagnostic procedure of brain death was not performed except for organ donation, which did not occur for these enrolled patients, so even with the patients entering a condition clinically equivalent to brain death, the treatment was continued until the heart beat stopped. For the patients whose families requested no treatment with a ventilator, even at blood pressure reduction, vasopressor infusion was not conducted.

### Evaluated Parameters

The primary outcome is in-hospital mortality. Possible factors affecting the mortality were analyzed and grouped into 3 categories, which were the pre-admission parameters, the clinical parameters during coma and the initial radiological parameters at entering the coma state. The pre-admission parameters included the following 5 factors: age at admission, sex, the pre-onset ADL expressed by modified Rankin Scale (mRS), current oral antiplatelet and/or anticoagulation treatment and receiving regular dialysis. The clinical parameters during coma included the following 3 factors: motor subset on GCS (GCS-M) at the coma diagnosis (eye and verbal response were both at 1 for these enrolled patients), abnormalities of pupils at the coma diagnosis, and the further presence of respiratory distress within 5 days of onset. The initial radiological parameters at entering the coma state included the following 6 factors: locations of hematoma categorized into the supratentorial (right and left) or infratentorial (brain stem and cerebellum), the existence of midline shift, intraventricular hemorrhage, brain stem compression, and hematoma on brain stem, and the maximum diameter of hematoma on head CT scan (the IVH was not included in the hematoma size).

Another evaluation was performed on the efficacy of the ventilator, analyzed among the patients manifesting the respiratory distress. The duration of respiratory distress was calculated from the medical records, between the first description on the medical records regarding respiration abnormalities and death or recovery from respiratory distress. Recovery from respiratory distress was defined as regular respiration rhythm for patients treated without a ventilator and weaning from the ventilator for patients treated with a ventilator. Respiratory distress was defined as apnea longer than 10 seconds or ataxic breathing. In order to extract respiratory distress only from brain stem dysfunction due to ICH compression, respiratory distress occurred within 5 days after admission was counted, but respiratory distress resulting from other etiologies such as pneumonia seen at the later period was not extracted. Endotracheal intubation itself was not counted as respiratory distress, since it was often only for airway clearance. Between the two groups supported by a ventilator (the ventilator group) and not supported by a ventilator (the no-ventilator group), survival duration and in-hospital mortality were compared.

All the surviving patients were followed up after discharge until October 31, 2013. Consciousness state at the last follow-up was also obtained by referral to the physician in charge. Disturbed consciousness was divided into 3 categories. Vegetative state (VS) is unresponsive but wakeful, and minimally conscious state (MCS), originally defined by Giacino et al. in 2002 [9], is awake and aware. The better state was described by Schnakers et al. in 2009 [10] as emerged from MCS (EMCS), judged with functional communication and/or functional object use at JFK Coma Recovery Scale-Revisited [11]. In making a referral to each physician who is taking care of the patient, the meaning and the difference in the above-mentioned 3 categories was ensured. In order to grasp how much improvement can occur for these patients after discharge, we also categorized the consciousness state of these surviving patients at discharge of our hospital in the same manner and set the tentative neurological condition at discharge although this assignment was used only for comparison with the final outcome.

### Statistical analysis

The 13 parameters mentioned above, possibly affecting the in-hospital mortality were analyzed univariately, then significant factors on univariate analysis were included in further multivariate analysis. The categorical parameters were evaluated using Chi-square analysis as the univariate, and the ordinal parameters (age, the pre-admission mRS, GCS-M and the maximum size of hematoma) were analyzed using Mann-Whitney U test as the univariate. Then significant parameters with these univariate analyses were included in the multivariate logistic regression model, to confirm the independently significant parameters on the in-hospital mortality.

For analysis of the ventilator effects on the prognosis, patients manifesting respiratory distress (56 patients) were examined. Both survival duration and in-hospital mortality were compared between the ventilator group (13 patients) and the no-ventilator group (43 patients). In-hospital mortality was compared with a contingency table using Chi-square analysis. The survival duration of the two groups was compared by using Man Whitney U test. Additionally, the mean survival duration was estimated using Kaplan-Meier method and the difference was compared by log-rank test. Results were considered statistically significant at *P*<0.05, and all *P* values were 2 sided. All patients data used for the analysis were shown in Appendix S1, 1–4.

## Results

### Overall outcome

**Figure 1:**
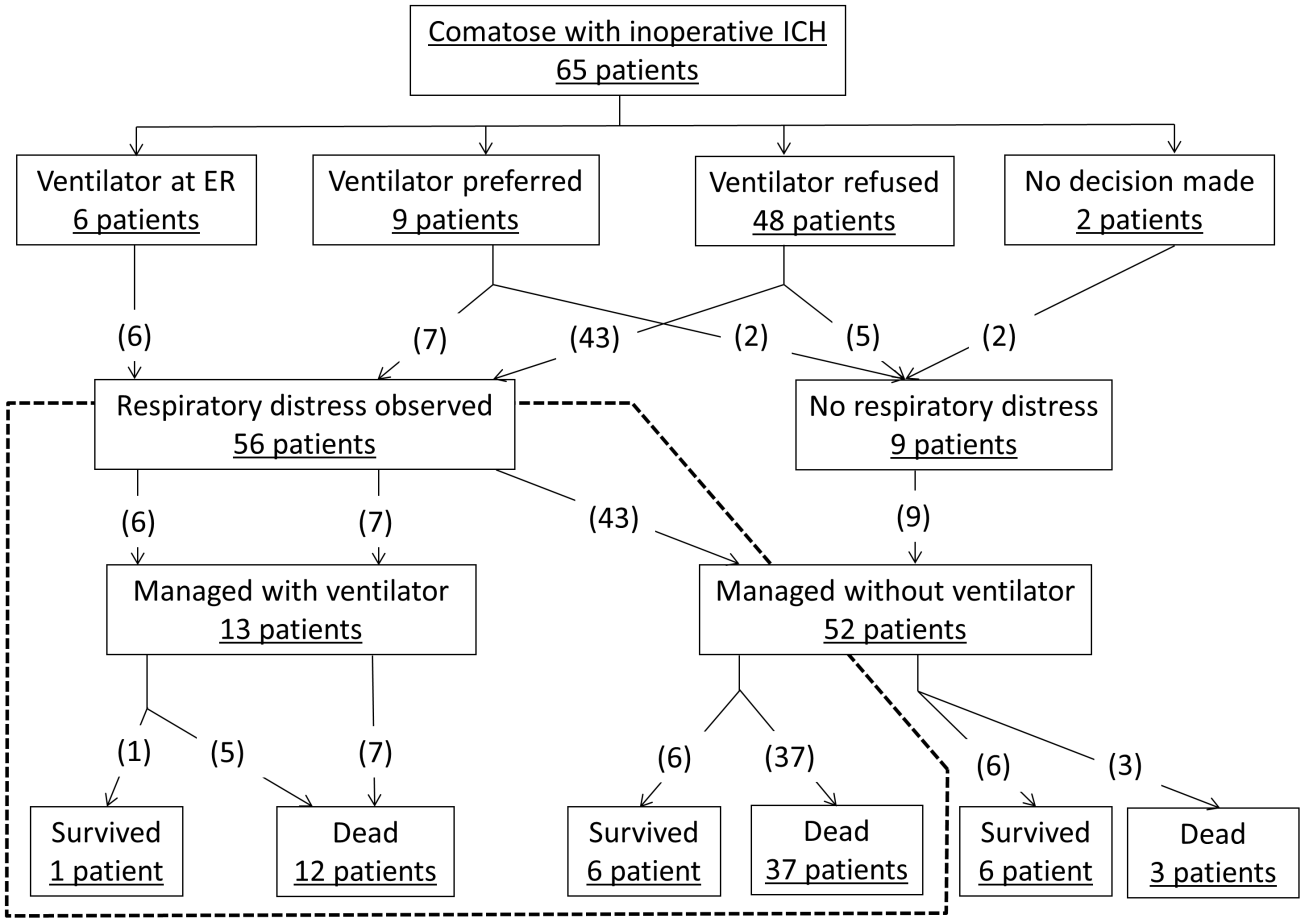
Flow diagram detailing the in-hospital mortality of the enrolled patients in relation to respiratory distress and ventilator usage. Enclosed area with dotted line indicates the outcome of the patients manifesting respiratory distress.

Among the enrolled 65 patients, 50 patients died due to ICH, and 2 patients were considered to have died with complications (pneumonia and myocardial infarction), so the gross in-hospital mortality was calculated as 80%. There were 13 patients who survived to discharge; however, all patients were severely disabled at 5 on mRS. Figure 1 shows overall mortality in relation to the occurrence of respiratory distress and ventilator usage.

### Parameters affecting the in-hospital mortality

With the univariate analysis, there were 6 factors affecting the in-hospital mortality. Using chi-square analysis, abnormal pupillary reflex (*P* = 0.001), respiratory distress (*P*<0.001), IVH (*P* = 0.003) and brain stem compression (*P* = 0.021) tended to be seen more frequently in the mortality group. Using Mann-Whitney U test, GCS-M tended to be low (*P*<0.001) and the maximum diameter of hematoma tended to be larger (*P* = 0.047) in the mortality group. Multivariate analysis was performed with these 6 factors, and only GCS-M at the coma diagnosis turned out to be a significant factor independently affecting the mortality (OR 3.1, *P* = 0.015), meaning the mortality increases approximately three-fold with each reduction of the GCS-M score. Details of the effects of the examined factors on mortality are shown in Table 1.

**Table 1:** Clinical characteristics of sixty-five enrolled comatose patients with inoperative ICH.

Factors	Survived (n = 13)	Dead in hospital (n = 52)	Univariate Analysis *		Multivariate Analysis
			*P* Value	OR (95% CI)	*P* Value
Pre-admission Parameters					
Age, years (mean)	58.2–96.7 (79.7)	46.7–94.2 (77.2)	0.47		
Female	7	23	0.53		
Pre-onset mRS (median)	0–5 (2)	0–5 (1)	0.20		
Pre-onset anti-platelet and/or anti-coagulation medications	5	25	0.53		
On dialysis	1	5	0.83		
Clinical Parameters					
GCS-M (median)	2–5 (4)	1–5 (2)	<0.001#	3.1 (1.2–7.8)	0.015#
Abnormal pupillary reaction	7	48	0.001#	5.6 (0.49–63.9)	0.17
Respiratory distress	7	49	<0.001#	4.9 (0.39–60.8)	0.22
Radiological Parameters					
Supratentorial	11	42	0.75		
Midline shift	5	34	0.076		
Intraventricular hemorrhage	6	44	0.003#	2.4 (0.29–19.6)	0.42
Brain stem compression	8	46	0.021#	3.3 (0.21–50.8)	0.39
Hematoma on brain stem	7	31	0.71		
Maximum diameter of hematoma, cm (mean)	3.0–7.6 (5.1)	1.0–9.5 (6.2)	0.047#	0.55 (0.28–1.1)	0.077

*Chi-square analysis for the categorical parameters, and Mann-Whitney U test for the ordinal parameters.

# Denotes statistical significance.

### Ventilator effect on prognosis

**Figure 2:**
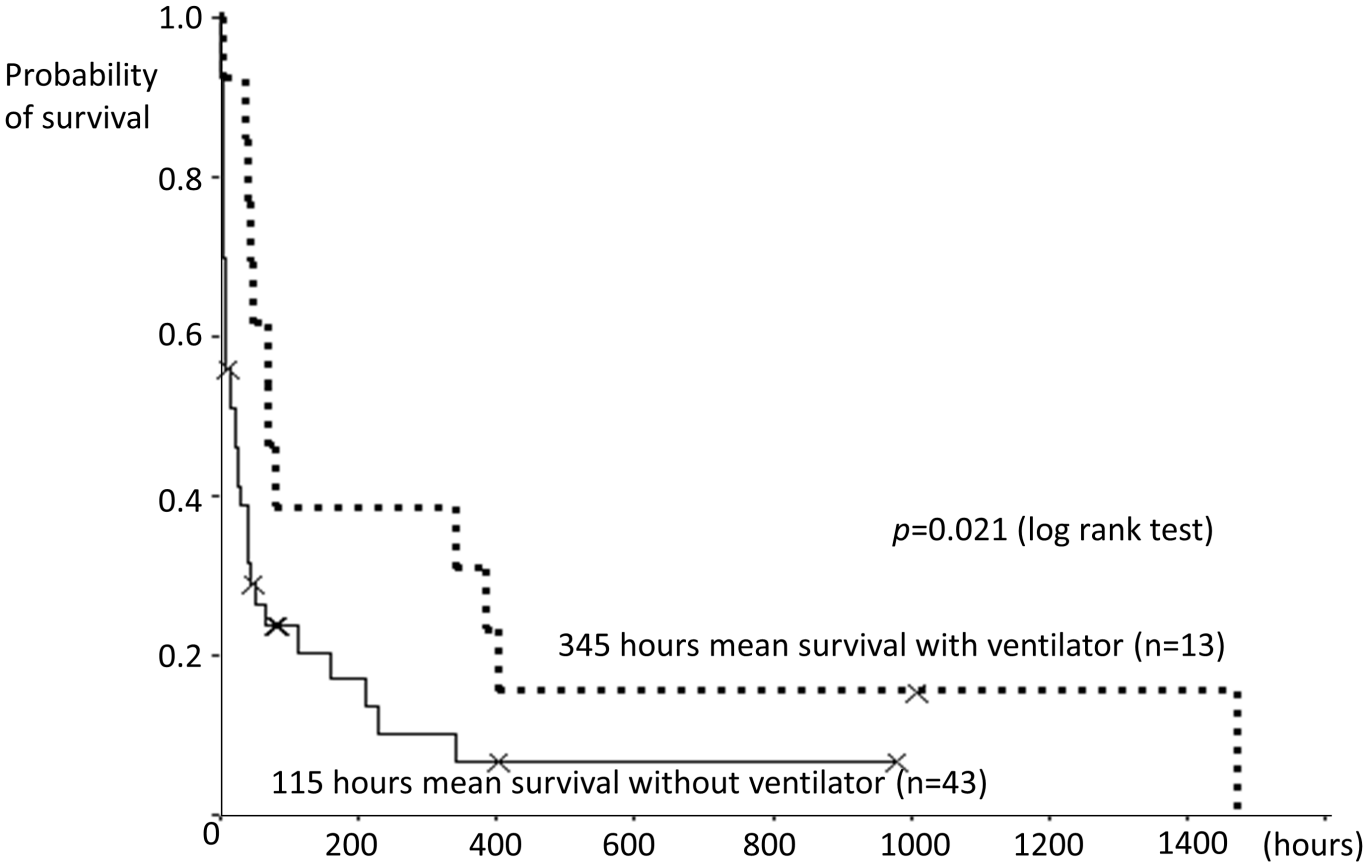
Graph of Kaplan-Meier survival curves after manifesting respiratory distress. The patients treated with the ventilator have longer survival duration.

The enclosed area with a dotted line in Fig. 1 indicates the overall mortality of the patients manifesting respiratory distress in relation with ventilator usage. With 56 patients who manifested respiratory distress, the effect of a ventilator was evaluated. Thirteen patients were treated with a ventilator (the ventilator group), due to the families' preference in 7 patients and due to resuscitation at ER in 6 patients, on the contrary 43 patients who did not have a ventilator (the no-ventilator group) were all due to the families' wishes. In all patients in the ventilator group, the ventilator was initiated within 12 hours after ICH onset. Among the 13 patients in the ventilator group, only 1 patient (7.7%) was successfully weaned from the ventilator and survived to discharge. Among 43 patients in the no-ventilator group, 6 patients (14.0%) survived to discharge. The contingency table about the in-hospital mortality in both groups was compared by Chi-square test, and no significant frequency deviation was observed (*P* = 0.72). The survival duration in the ventilator group was 309.1±447.2 hours (mean ± standard deviation), which is significantly longer than that in the no-ventilator group (73.7±166.9 hours, *P* = 0.001) by using Man Whitney U test. The survival durations were also estimated using Kaplan-Meier method and the differences between the two groups were compared with log-rank test (Fig. 2). Again, the mean survival duration in the ventilator group (345 hours) was significantly longer than that in the no-ventilator group (115 hours, *P* = 0.021). The ventilator has an impact on prolonging survival duration although no effect was observed on in-hospital mortality.

### Outcome of the surviving patients

Thirteen patients survived and were discharged from our hospital in the studied cohort. They were followed up for 5 to 50 months (mean 18.2 months). Their consciousness levels at discharge were VS in 8 patients, MCS in 2 patients and EMCS in 3 patients. Among these patients, 7 patients died at a nursing hospital between 5 and 29 months from onset. The most frequent cause of death was pneumonia (4 among 7 patients). No patients had better consciousness than EMCS, indicating all surviving patients suffered from continuously-disabled life due to prolonged consciousness disturbances. Although severely disturbed cognition remained for all patients, 4 patients had improvement in consciousness (VS to MCS in 2 patients, MCS to EMCS in 1 patient and VS to EMCS in 1 patient). On the contrary, 4 patients deteriorated after discharge (EMCS to MCS in 3 patients and MCS to VS in 1 patient), and another 5 patients were unchanged and remained at VS. The details of the surviving patient outcome are shown in Table 2.

**Table 2:** Clinical characteristics and outcome of surviving patients.

Case No.	Age/Sex	Hematoma location	GCS (motor)	Diameter of hematoma (cm)	Respiratory distress	Ventilator used	Consciousness at discharge (days from onset)	Consciousness at last follow-up	Final survival/Months from onset (cause of death)
1	88.4/F	Lt supra	4	3.2	none	(no need)	MCS (25 days)	VS	alive/50 months
2	69.9/M	Lt supra	4	6.7	none	(no need)	VS (10 days)	EMCS	dead/24 months (renal failure)
3	81.5/F	Lt supra	5	6.9	observed	no	EMCS (97 days)	MCS	dead/6 months (pneumonia)
4	92.9/F	Rt supra	5	4.9	observed	no	VS (44days)	VS	dead/14 months (pneumonia)
5	76.3/M	Rt supra	4	5.0	none	(no need)	EMCS (46 days)	MCS	alive/25 months
6	80.0/M	Rt supra	3	5.2	observed	no	VS (67 days)	VS	alive/23 months
7	96.7/F	Lt supra	4	6.0	none	(no need)	EMCS (38 days)	MCS	dead/14 months (unspecified)
8	76.7/M	Lt supra	4	3.0	none	(no need)	VS (54 days)	VS	dead/29 months (pneumonia)
9	59.9/F	Rt supra	4	4.0	observed	no	MCS (60 days)	EMCS	alive/10 months
10	58.2/M	Lt supra	2	6.4	observed	yes	VS (105 days)	VS	alive/9 months
11	87.7/F	BS	4	3.8	observed	no	VS (84 days)	VS	alive/8 months
12	85.7/F	BS	5	3.1	observed	no	VS (29 days)	MCS	dead/5 months (unspecified)
13	82.1/M	Lt supra	5	7.6	none	(no need)	VS (64 days)	MCS	dead/20 months (pneumonia)

BS = brain stem; EMCS = emerged from minimally conscious state; MCS = minimally conscious state; supra = supratentorial; VS = vegetative state.

## Discussion

It is the primary responsibility of physicians to pursue the best possible outcome for the patients; however, the debate over medical futility in order to control medical costs or decrease the burden on the medical resources has been a major topic [12], [13], and the advances in medical technologies unfortunately exaggerates the costly life-sustaining [12]. A conflicting mission, determining low-priority care concurrently with pursuing the better outcome, is imposed on physicians in the current “medico-social triage” era, to keep the medical care system functioning properly. Establishing concise outcome prediction models depending on the various patient presentations is thus desired. Although the guidelines for managing ICH have been revised [8], the role of hematoma evacuation is uncertain [7], [14]. We still believe that hematoma evacuation surgery should not be abandoned as a futile treatment since many patients who are able to return to their original lives with only limited disabilities [15]. Neurosurgeons should clarify the treatment strategies taking into account the complications of the patients' conditions, including age, etiology, lesional location and volume, consciousness levels, neurological deficits at presentation and comorbid systematic diseases [14].

Abnormal respiratory patterns are well-known symptoms for the comatose patients with ICH [16], and many comatose patients manifest respiratory distress either at presentation or in the early period after admission. Surgical evacuation of hematoma may play a role as the last resort; however, the operations often cannot be attempted due to the associated conditions of the patients, including the brain stem location of hematoma, or other comorbid significant diseases. Few options are left for these patients, and the role of artificial ventilator support may be the only the debatable issue; however, there is no description in the guidelines regarding respiration support [8]. Elliott et al. mention, “airway management, including endotracheal intubation and mechanical ventilation, is a priority in the unconsciousness patient or in those with a deteriorating conscious level” in their review article [17], although no indication of ventilation is described. The mortality rate for ventilated patients with stroke was once reported as from 57 to 90% [18], however, indications using the ventilator seem to differ between institutes. Gujjar et al. described ventilation indicated for all intubated patients [18]. In a recent article by Elmer et al., among 697 ventilated patients, 17% of the patients were with GCS score between 13 and 15, and only 20% of the patients had hypoxemia [19]. In our institute, ventilator usage for the management of patients with ICH was mostly limited for patients manifesting respiratory distress, and the studied patients were more uniform than previously reported, that is E1V1at GCS score (3–7 in total) due to inoperative ICH. Our retrospective study revealed that patients in such a specifically broken-down group would not receive significant benefits from treatment with a ventilator. The significance of this study may be specific to Japan where even brain death patients are cared for with a ventilator, and the results of this study will not be attractive at all for most western clinicians since the patients in the current study may be considered as contraindicating ventilator usage as “too much”, and DNR orders for these patients will not lead to a self-fulfilling prophecy [1]–[4], since their medical conditions were so critical. However, it should be noted that one-third of neurologically ill patients requiring a ventilator (mixtures of different conditions) recovered fairly well (better than mRS4) [6], and self-fulfilling prophecy can be a problem in many cases with ICH. We should be clear about the danger of self-fulfilling prophecies, and the proper aggressive treatment, including the ventilator usage, and surgical intervention is to be considered for a significant amount of patients with ICH, although the self-fulfilling prophecies are not an issue for our studied subgroup.

There are several limitations in the current study. Since our study was based on a small number of patients; only 13 patients managed with ventilators, it may be too soon to draw a conclusion. However, in the comparison of the patients treated without ventilators, the survival rate did not improve at all; actually the rate was even worse (7.7% in the ventilator group versus 14.0% in the no-ventilator group), and we believe this result is worthy of remark even with the small numbers. Secondary, the policy for the management of critically-ill patients with inoperative ICH significantly differs between countries, hence, the present investigation is not necessarily extendible to the centers in other countries. In addition, there are several limitations resulting from the retrospective method. Due to the lack of prospectively-set survey sheets, pre-existing comorbidities, which should have been analyzed as the pre-admission parameters, could not be uniformly obtained, and thus were not included. As another possible limitation inherent to retrospective methods, due to the insufficient diagnostic procedures at admission for the inclusion/exclusion determination, several patients to be excluded were difficult to define from medical charts at admission, i.e., the patients manifesting epileptic respiratory arrest or abnormal respiration due to pulmonary embolism with small hematoma should be treated with a ventilator, and they are to be excluded from the current study, because their ventilator usage was medically required. Nevertheless, it was not a difficult task to distinguish respiratory distress due to ICH from that due to other etiologies by evaluating the medical course. We believe the proper inclusion/exclusion was performed in this study.

Our results indicate the outcome of the patients with inoperative ICH was uniformly poor, with 80% in-hospital mortality, and even surviving patients had severely disturbed cognition. Based on these results, treatments for comatose patients with inoperative ICH may be regarded as medically futile. However, when determining the futility of medical care, the ethical clarification regarding a “meaningful” life is prerequisite. The definition of a life worth saving by spending public costs varies among countries and may change over time even in the same country. The social attitude against the acceptance of brain death is an evident example. Although brain death is world-widely accepted concept [20], the policy on when to termination treatment with a ventilator after the diagnosis of brain death differs between countries and even between states in the US [21]. Brain death is still not accepted as “general” death in Japan, which is only diagnosed when organ donation for transplantation is attempted [22]. The withdrawal of mechanical ventilation unlikely occurs in Japan even after the confirmation of brain death, although initial refusal of ventilator support is frequently seen. And once managed with a ventilator, the clinical confirmation of brain death will not be attempted even for “clinically-assumed brain death”. Among 12 patients who eventually died after being managed with the ventilator, we believe most patients resulted in the undiagnosed brain death during the course of the treatment, but medical care was continued for days. This may result from the special attitude of Japanese to death [22]: life-sustaining of brain-dead patients is not necessarily meaningless for many Japanese people [23], and such an attitude of the patient's family may be seen even in the US [24]. However, the burden on medical resources by prolonging a brain-dead patient's life is so significant and the termination of medical care for brain-dead patients should be socially accepted when establishing the guideline for the medico-social triage, otherwise no medical treatment can be judged as generally “futile”.

In order to decrease the burden on public medical resources and to maintain the proper function of the healthcare system, a reasonable boundary line of medical futility should be established, and we believe that the treatment for patients fulfilling the criteria of futility should be abandoned uniformly and fairly. Study to determine futile medical treatments should be recognized as medico-socially worthwhile. In many outcome studies in neurological fields, “dependent” is often seen as the boundary of a poor outcome [25], [26]. Although the functional outcome; whether dependent or independent in the future is definitely a necessary observation, this boundary is not appropriate for medical futility since many dependent patients are capable of expressing their will. In fact, without the patients' advanced directives, medical care termination decided by the families because of the certainty of a functionally-dependent outcome, is ethically problematic. We propose “treatment resulting in the irreversible severely-disturbed consciousness at best” as a medical futility, and considering the perspective of this proposal, the consciousness level should be more sophisticatedly evaluated. In this report, the outcome for surviving patients was segmentalized according to their consciousness levels, which were VS, MCS and EMCS. No individual physician can define which level of the outcome is medically futile so far, and thorough national debate in each country is to be encouraged in order to establish the guidelines on how to restrict the medical care for patients with severely-disturbed consciousness. And in this debate, the recognition of the consciousness improvement, occasionally remarkable ones after a certain period [27], is essential. In fact, consciousness improvement was observed in 4 patients among our 13 surviving patients, although no patients became better than EMCS. More detailed analysis in respect of the type of the injury, the severity of the consciousness disturbance, the age of the patients and the possible expected recovery after a certain period, at least 12 months, will be needed for determining medical futility.

## Conclusions

Even for neurosurgeons, determining medical futility is essential with the limited medical resources, and ventilator usage for comatose patients with inoperative ICH should be reconsidered. Further studies should be conducted with patients who have undergone hematoma evacuation for their ICH to confirm surgeries to be avoided. A reasonable boundary for medical futility should be considered in relation to the consciousness level, irrespective of functional disabilities. Detailed analysis of the consciousness outcome should be included in further studies relating to medical futility.

## Supporting Information

Appendix S1 **Appendix 1–4.**
(DOCX)
