# Supplementary material for: Mechanical Ventilation for Comatose Patients with Inoperative Acute Intracerebral Hemorrhage: Possible Futility of Treatment
Source: PLoS One. 2014 Jul 25;9(7):e103531. doi: 10.1371/journal.pone.0103531 (PMC4111623; doi:10.1371/journal.pone.0103531)
Supplement: Appendix S1 — Appendix 1–4. (DOCX) [file pone.0103531.s001.docx]

**Appendix 1 Pre-admission parameters of all analyzed patients**

Case Age Sex Pre-onset Anti-platelet or On dialysis

No. mRS coagulation medications

Surviving patients at discharge

1 88.4 Female 4 No No

2 69.9 Male 2 No Yes

3 81.5 Female 3 Yes No

4 92.9 Female 1 No No

5 76.3 Male 0 No No

6 80.0 Male 0 No No

7 96.7 Female 3 Yes No

8 76.7 Male 3 Yes No

9 59.9 Female 3 No No

10 58.2 Male 5 No No

11 87.7 Female 1 Yes No

12 85.7 Female 0 No No

13 82.1 Male 1 Yes No

Patients leading to mortality

14 85.3 Male 0 Yes No

15 79.1 Male 0 No No

16 90.7 Female 2 No No

17 60.0 Male 1 Yes Yes

18 71.4 Male 1 Yes Yes

19 56.2 Female 0 Yes Yes

20 73.3 Male 1 Yes Yes

21 76.3 Female 3 No Yes

22 81.0 Female 0 Yes No

23 85.5 Female 4 Yes No

24 82.3 Female 2 Yes No

25 80.9 Male 3 Yes No

26 64.9 Female 0 Yes No

27 76.5 Male 0 Yes No

28 79.3 Male 3 Yes No

29 82.3 Male 0 Yes No

30 79.3 Male 2 Yes No

31 83.3 Male 0 Yes No

32 78.9 Female 3 Yes No

33 87.7 Female 3 Yes No

34 83.8 Female 0 Yes No

35 79.0 Male 1 Yes No

36 70.1 Female 3 Yes No

37 77.4 Female 1 Yes No

38 86.5 Male 1 Yes No

39 87.9 Female 3 Yes No

40 87.8 Male 2 Yes No

41 65.8 Female 0 Yes No

42 67.1 Male 2 No No

43 75.0 Male 0 No No

44 83.7 Male 0 No No

45 83.9 Female 3 No No

46 83.5 Male 2 No No

47 79.3 Male 1 No No

48 46.7 Male 0 No No

49 86.1 Male 5 No No

50 67.8 Male 4 No No

51 57.1 Male 0 No No

52 83.7 Female 5 No No

53 57.8 Male 0 No No

54 91.4 Male 3 No No

55 55.5 Male 0 No No

56 88.9 Female 3 No No

57 80.4 Female 0 No No

58 90.6 Female 2 No No

59 72.3 Female 1 No No

60 78.3 Female 0 No No

61 91.8 Female 0 No No

62 60.8 Female 0 No No

63 94.2 Male 1 No No

64 78.6 Male 0 No No

65 66.8 Male 2 No No

**Appendix 2 Clinical parameters during coma of all analyzed patients**

Case GCS-M Abnormalities Respiratory

No. of pupils distress

Surviving patients at discharge

1 4 No none

2 4 No none

3 5 Yes observed

4 5 No observed

5 4 Yes none

6 3 Yes observed

7 4 No none

8 4 No none

9 4 Yes observed

10 2 No observed

11 4 Yes observed

12 5 Yes observed

13 5 Yes none

Patients leading to mortality

14 2 Yes none

15 3 Yes none

16 5 No none

17 2 Yes observed

18 4 Yes observed

19 4 Yes observed

20 2 No observed

21 1 Yes observed

22 4 Yes observed

23 2 Yes observed

24 2 Yes observed

25 2 Yes observed

26 1 Yes observed

27 2 Yes observed

28 4 Yes observed

29 2 Yes observed

30 4 Yes observed

31 1 Yes observed

32 4 Yes observed

33 4 Yes observed

34 2 Yes observed

35 1 Yes observed

36 4 Yes observed

37 1 Yes observed

38 1 Yes observed

39 2 Yes observed

40 1 Yes observed

41 4 Yes observed

42 2 Yes observed

43 2 Yes observed

44 4 Yes observed

45 2 Yes observed

46 1 Yes observed

47 2 Yes observed

48 2 Yes observed

49 1 Yes observed

50 2 Yes observed

51 1 Yes observed

52 1 Yes observed

53 4 Yes observed

54 4 Yes observed

55 1 Yes observed

56 1 Yes observed

57 3 Yes observed

58 4 Yes observed

59 2 Yes observed

60 1 Yes observed

61 4 Yes observed

62 2 Yes observed

63 4 Yes observed

64 2 No observed

65 1 No observed

**Appendix 3 Radiological parameters at entering the coma state of all analyzed patients**

Case Location of Midline Intraventricular Brain stem Hematoma on Diameter of

No. hematoma shift hemorrhage compression brain stem hematoma

Surviving patients at discharge

1 left supratentorial No No Yes Yes 3.2

2 left supratentorial No No No No 6.7

3 left supratentorial Yes No No No 6.9

4 right supratentorial Yes Yes Yes No 4.9

5 right supratentorial Yes Yes Yes Yes 5.0

6 right supratentorial Yes Yes Yes Yes 5.2

7 left supratentorial No No No No 6.0

8 left supratentorial No Yes Yes Yes 3.0

9 right supratentorial No No Yes Yes 4.0

10 left supratentorial No No No No 6.4

11 brain stem No Yes Yes Yes 3.8

12 brain stem No No Yes Yes 3.1

13 left supratentorial Yes Yes No No 7.6

Patients leading to mortality

14 brain stem No Yes Yes Yes 4.0

15 right supratentorial Yes Yes Yes Yes 8.4

16 left supratentorial No Yes No No 9.0

17 right supratentorial Yes Yes Yes Yes 5.7

18 right supratentorial Yes Yes Yes Yes 5.9

19 left supratentorial Yes Yes Yes Yes 5.3

20 right supratentorial Yes Yes No No 8.6

21 left supratentorial Yes Yes Yes Yes 7.0

22 brain stem No Yes Yes Yes 4.8

23 right supratentorial Yes Yes Yes Yes 8.5

24 right supratentorial Yes Yes Yes Yes 7.4

25 right supratentorial Yes Yes Yes Yes 5.7

26 left supratentorial Yes Yes Yes Yes 6.3

27 left supratentorial Yes Yes Yes Yes 1.0

28 left supratentorial Yes Yes Yes Yes 7.1

29 left supratentorial Yes Yes Yes Yes 3.0

30 left supratentorial Yes Yes Yes Yes 9.0

31 brain stem No Yes Yes Yes 3.4

32 right supratentorial Yes Yes Yes No 7.9

33 right supratentorial Yes Yes Yes No 9.0

34 right supratentorial Yes Yes Yes No 7.7

35 right supratentorial Yes Yes Yes No 9.5

36 left supratentorial Yes Yes Yes No 7.1

37 cerebellum No Yes Yes No 4.2

38 cerebellum No Yes Yes No 5.0

39 right supratentorial Yes No Yes No 6.3

40 right supratentorial No Yes No No 4.6

41 right supratentorial Yes No No No 6.8

42 right supratentorial Yes Yes Yes Yes 9.5

43 right supratentorial Yes Yes Yes Yes 7.9

44 right supratentorial Yes Yes Yes Yes 6.3

45 left supratentorial Yes Yes Yes Yes 6.2

46 left supratentorial Yes Yes Yes Yes 7.0

47 left supratentorial Yes Yes Yes Yes 6.2

48 left supratentorial Yes Yes Yes Yes 7.4

49 left supratentorial No Yes Yes Yes 4.3

50 cerebellum No Yes Yes Yes 6.4

51 brain stem No Yes Yes Yes 4.8

52 right supratentorial No No Yes Yes 4.4

53 right supratentorial No No Yes Yes 3.1

54 brain stem No No Yes Yes 2.3

55 brain stem No No Yes Yes 4.1

56 right supratentorial Yes Yes Yes No 5.2

57 left supratentorial Yes Yes Yes No 7.2

58 left supratentorial Yes Yes Yes No 7.8

59 left supratentorial Yes Yes Yes No 7.3

60 right supratentorial No Yes Yes No 5.1

61 left supratentorial No No Yes No 5.6

62 right supratentorial Yes Yes No No 7.3

63 right supratentorial No Yes No No 5.7

64 brain stem No Yes Yes Yes 3.3

65 right supratentorial Yes No Yes No 8.3

**Appendix 4 Ventilator usage and duration of all analyzed patients with respiratory distress**

Case Ventilator Duration of

No. used respiratory distress (hours)

3 No 79

4 No 10

6 No 82

9 No 48

10 Yes 1008

11 No 979

12 No 403

17 No 24

18 No 20

19 No 5

20 No 7

21 No 2

22 Yes 1474

23 No 23

24 No 227

25 No 7

26 Yes 70

27 Yes 342

28 No 5

29 No 41

30 No 66

31 No 210

32 No 50

33 No 4

34 Yes 81

35 No 5

36 No 5

37 Yes 38

38 Yes 404

39 No 41

40 No 1

41 No 4

42 No 2

43 Yes 44

44 No 1

45 No 28

46 No 9

47 No 7

48 Yes 43

49 No 39

50 Yes 50

51 Yes 7

52 No 114

53 No 15

54 No 24

55 No 9

56 No 340

57 No 1

58 No 9

59 No 44

60 Yes 72

61 No 161

62 Yes 385

63 No 2

64 No 3

65 No 15
